# Effectiveness of physical therapy techniques for cancer-related pain: a systematic review

**DOI:** 10.3389/fresc.2026.1680725

**Published:** 2026-02-02

**Authors:** Iser Alejandro González-Ramírez, Xavier Oswaldo Molina-López, Leonardo Enrique Bermeo-Gualán, Nuria Bonsfills-García, Elena Velarde-Fernández, Vanesa Abuín-Porras

**Affiliations:** 1 Universidad Europea de Madrid, Faculty of Medicine, Health and Sports, Campus de Villaviciosa, Calle Tajo s/n. 28670 Villaviciosa de Odón, Madrid, Spain; 2 STRONG Research Group, Universidad Europea de Madrid, Faculty of Medicine, Health and Sports, Madrid, Spain

**Keywords:** cancer survivorship, electrotherapy, exercise therapy, oncologic rehabilitation, pain management

## Abstract

**Background:**

Cancer-related pain is a frequent and disabling symptom that negatively affects function and quality of life. Physiotherapy interventions are increasingly used as adjuvant treatments to alleviate pain and improve functional recovery in oncology patients.

**Objective:**

To evaluate the scientific evidence on the effectiveness of physiotherapy interventions in reducing cancer-related pain and improving functional outcomes.

**Methods:**

A systematic review was conducted following PRISMA 2020 guidelines (PROSPERO ID: CRD42026542801). Searches were performed in PubMed, Cochrane, CINAHL, and PEDro databases between January 30 and February 15, 2025, including randomized controlled trials published in English or Spanish with PEDro scores ≥ 6.

**Results:**

Eight randomized controlled trials published between 2020 and 2024 met the inclusion criteria, encompassing 514 participants. Interventions included resistance and aerobic exercise, sensorimotor training, electrotherapy, and multimodal rehabilitation programs. Most studies reported significant reductions in pain intensity, improvements in functional capacity and quality of life, and no serious adverse effects. The methodological quality of the included trials was moderate to high.

**Conclusions:**

Physiotherapy interventions, particularly structured exercise and electrotherapy, appear to be effective and safe adjuvant strategies that may contribute to improvements in pain-related and functional outcomes in people with cancer. The available evidence predominantly addresses neuropathic pain associated with chemotherapy-induced peripheral neuropathy. Nevertheless, heterogeneity among protocols and small sample sizes limit the strength of conclusions, underscoring the need for additional high-quality randomized controlled trials.

**Systematic Review Registration:**

PROSPERO CRD42026542801.

## 1 Introduction

Cancer-related pain represents one of the most frequent and limiting clinical manifestations among people living with this disease. Despite advances in oncologic treatment, pain continues to be a prevalent symptom in all phases of the care process: from diagnosis, during active treatment and in the survivorship stage, to palliative care (1). In recent years, several novel pharmacological approaches have shown promising results for cancer-related pain, including the use of tetrodotoxin as a sodium channel blocker (2), perineural and regional nerve block techniques (3), and the recent development of suzetrigine, a selective NaV1.8 inhibitor, currently in clinical evaluation for neuropathic and oncologic pain (4).The scientific literature provides evidence that this pain not only affects the patient physically, but significantly interferes with their quality of life, psychological well-being and overall functionality (5, 6).

Cancer-related pain remains one of the most frequent and disabling symptoms throughout the oncologic continuum—from diagnosis and active treatment to survivorship and palliative care (1–3, 7–10). It is estimated that around 50% of patients receiving active treatment and more than 70% of those with advanced cancer experience clinically significant pain, with prevalence varying by tumor type (1, 7–11). Pain may affect approximately 40% of patients with breast or lung cancer (10) and up to 42% of those with head and neck malignancies (12), underscoring the need for comprehensive and multidisciplinary management strategies.

Within the spectrum of cancer-related pain, chemotherapy-induced peripheral neuropathy (CIPN) represents one of the most disabling and prevalent complications, affecting up to 40% of patients treated with agents such as taxanes, platinums or vinca alkaloids (13, 14). This condition manifests with symptoms such as paresthesias, burning pain and loss of balance, negatively impacting the patient's quality of life and functionality (15). Although pharmacological options are limited, physical therapy has emerged as a promising intervention. Recent systematic reviews have shown that physical exercise programs can alleviate CIPN symptoms and improve patients' quality of life (9, 16). However, the need for further research is highlighted due to the heterogeneity of interventions and small sample sizes in existing studies.

These findings highlight the need to improve pain management strategies from an interdisciplinary perspective. In particular, physical therapy has emerged as a promising tool within the nonpharmacologic approach (17). While pain care has historically focused on opioids, there are multiple barriers to their effective use, including fear of addiction, underdiagnosis by healthcare professionals, and poor patient-physician communication (18–20). In this context, the integration of physiotherapeutic techniques could not only reduce pain, but also improve mobility, quality of life, and the patient's perception of control over his or her disease (21).

According to the review by Evenepoel et al. (10), during active treatment, approximately 40% of patients report pain. This high prevalence reflects both the toxicity of treatments and the lack of standardization in the assessment and approach to pain. In addition, there is considerable variability depending on the type of cancer. In the case of head and neck tumors, for example, it has been documented that pain can start even before treatment and persist after its completion (12). Other studies have begun to explore the influence of physical activity on pain modulation in cancer patients, showing positive results, although still limited by methodological heterogeneity (22–26). While cancer-related pain encompasses a wide range of mechanisms, including nociceptive, neuropathic, and mixed pain syndromes, the current body of randomized evidence on physical therapy interventions is largely concentrated on neuropathic pain associated with CIPN. The present work arises from the need to synthesize the scientific evidence on the efficacy of various physical therapy techniques in the management of cancer-related pain. In oncologic rehabilitation research, terminology such as physiotherapy, physical therapy, exercise therapy, and electrotherapy is sometimes used interchangeably, which may obscure important conceptual distinctions between intervention types. In this review, interventions are categorized as exercise-based rehabilitation (structured and prescribed therapeutic exercise targeting physical function), electro-physical agents (non-invasive electrical stimulation modalities), or adjunctive neuromodulatory techniques (e.g., electroacupuncture or transcutaneous electrical acupoint stimulation). Although there are multiple clinical trials with favorable results, a comprehensive view has not yet been consolidated to identify the most effective techniques, the types of pain that are best treated and the characteristics of the patients who benefit most from these interventions. This review summarizes and critically analyzes the available evidence on physiotherapy interventions for cancer-related pain.

## 2 Objectives

The main objective of this review is to determine the efficacy of various physical therapy techniques for cancer-related pain.

The specific objectives consisted on: Record the Physiotherapy techniques used for cancer-related pain; Describe the effects of physical therapy techniques on cancer-related pain; Identify the most effective techniques for the management of cancer-related pain.

## 3 Methods

### 3.1 Study design

Literature review reviewed by IAGR and XOML. In case of disagreement LEBGL was consulted. This review was conducted between the months of February and March 2025, and prospectively registered in PROSPERO CRD42025642801. The last literature search was conducted on February 15, 2025, covering studies published between January 1, 2018, and February 15, 2025.

### 3.2 Search strategy

PubMed, Cochrane, CINAHL and PEDro databases were consulted, using MESH terms such as "physiotherapy, "physical therapy, "exercise therapy, "manual therapy, "rehabilitation, "oncological rehabilitation, "cancer rehabilitation, "cancer patients, "cancer survivors, "pain management, "lymphedema management, "fatigue management, "quality of life, "electrotherapy, "breathing exercises together with Boolean operators ("AND and "OR) Annex 1.

The search was limited to studies published within the last five years to ensure that the findings reflect current clinical practices and methodological standards in oncologic rehabilitation.

### 3.3 Selection criteria

#### 3.3.1 Inclusion criteria

Articles will be included if they meet the following conditions following the PICO question scheme.

##### 3.3.1.1 Population

Individuals diagnosed with any type of cancer who are undergoing or have undergone treatment.

Patients 18 years of age and older.

##### 3.3.1.2 Interventions

Participants must receive physical therapy as part of their oncologic rehabilitation, which may include interventions for physical recovery, functional restoration, pain management, fatigue, mobility, or management of lymphedema related to cancer or its treatment.

The intervention should be a physical therapy technique aimed at oncologic rehabilitation.

Interventions may be provided in isolation or as part of a multimodal physical therapy program. Non-pharmacological physiotherapy-based interventions were eligible if they could be clearly classified into one of the following categories: (1) exercise-based rehabilitation, defined as structured therapeutic exercise programs prescribed to improve physical function or neuromuscular performance; (2) electro-physical agents, including non-invasive electrical stimulation modalities such as transcutaneous electrical nerve stimulation; and (3) adjunctive neuromodulatory techniques, including electroacupuncture or transcutaneous electrical acupoint stimulation.

Studies should provide sufficient detail on the content and delivery of the intervention to allow for replication or assessment of applicability in clinical practice.

##### 3.3.1.3 Comparator

Comparator or control groups undergoing sham intervention, placebo, or no intervention.

Control groups receiving standard care, treatment as usual, or alternative physical therapy techniques that allow a direct comparison between two appropriate physical therapy methods.

Outcomes: Studies applying different physiotherapy techniques used in oncology rehabilitation such as therapeutic exercise, physical agents, manual techniques performed in clinical, community or home settings.

Study design: Randomized Controlled Trial.

#### 3.3.2 Exclusion criteria

In the same way, articles that do not meet the following conditions will be excluded:

Participants without a diagnosis of cancer.
Populations with other non-cancer primary conditions where their intervention is not related to oncologic rehabilitation.
Studies that only include pediatric or adolescent populations.
Studies where the intervention is primarily psychological, pharmacological or surgical without physical rehabilitation.
Participants undergoing Complementary, alternative medicine interventions with no recognised history or interventions that are not relevant to Physiotherapy.
The study does not describe the physical therapy intervention in sufficient detail to assess its nature and delivery.
Pilot or feasibility studies that do not measure outcomes related to efficacy.
Studies published more than 5 years ago.
Studies scoring lower than 6 points on the PEDRO scale.

### 3.4 Assessment of quality and risk of bias

The quality and risk of bias of included studies were assessed using the Cochrane Risk of Bias Tool (RoB) 2.0 for randomized controlled trials (Annex 1).

### 3.5 Qualitative analysis of the level of scientific evidence

Only studies with PEDro scores ≥ 6 were includedwere included (Table 1).

**Table 1:** Analysis of the methodological quality of the studies analyzed in this review, using the PEDro scale.

Items	Chan K, et al. (29)	He L, et al. (30)	Lu Z, et al. (31)	Lyu Z, et al. (32)
The election criteria were specified*	1	0	1	0
Subjects were randomly assigned to the following groups	1	1	1	1
Assignment was concealed	1	0	1	1
Groups were similar at baseline	1	1	1	1
All subjects were blinded	1	0	0	1
All therapists administering the therapy were blinded	0	0	0	0
All assessors who measured at least one key outcome were blinded	1	0	1	1
Measurements of at least one of the key outcomes were obtained from more than 85% of subjects	1	1	1	1
Results were presented for all subjects or intention-to-treat analysis.	0	1	1	1
Between-group comparisons were made on at least one variable	1	1	1	1
Study provides point-in-time and variability measures for at least one key outcome	1	1	1	1
Total	8/10	6/10	8/10	9/10
*Not evaluable item				
Items	Müller J, et al. (34)	Song SY, et al. (27)	Tian W, et al. (33)	Yu X, et al. 2024
The election criteria were specified*	1	1	1	1
Subjects were randomly assigned to the following groups	1	1	1	1
Assignment was concealed	0	1	0	1
Groups were similar at baseline	1	1	1	1
All subjects were blinded	0	1	0	0
All therapists who administered the therapy were blinded	0	1	0	0
All assessors who measured at least one key outcome were blinded	1	1	1	0
Measurements of at least one of the key outcomes were obtained from more than 85% of subjects	1	1	1	1
Results were presented for all subjects or intention-to-treat analyses	1	1	1	1
Comparisons were made between groups on at least one variable	1	1	1	1
Study provides point-in-time and variability measures for at least one key outcome	1	1	1	1
Total	7/10	10/10	7/10	7/10
*Not evaluable item				

## 4 Results

### 4.1 Flow diagram

A total of 638 records were identified through database searches (PubMed, Cochrane, CINAHL, and PEDro). After removing 84 duplicates, 554 records remained for title and abstract screening. Of these, 422 were excluded for not meeting the inclusion criteria. The full text of 132 articles was assessed in detail, and 124 were excluded for the following reasons: not randomized controlled trials (*n* = 58), not physiotherapy-based interventions (*n* = 36), no pain outcome (*n* = 24), and methodological quality below the PEDro threshold (*n* = 6). Finally, 8 studies met all eligibility criteria and were included in the qualitative synthesis.

**Figure 1:**
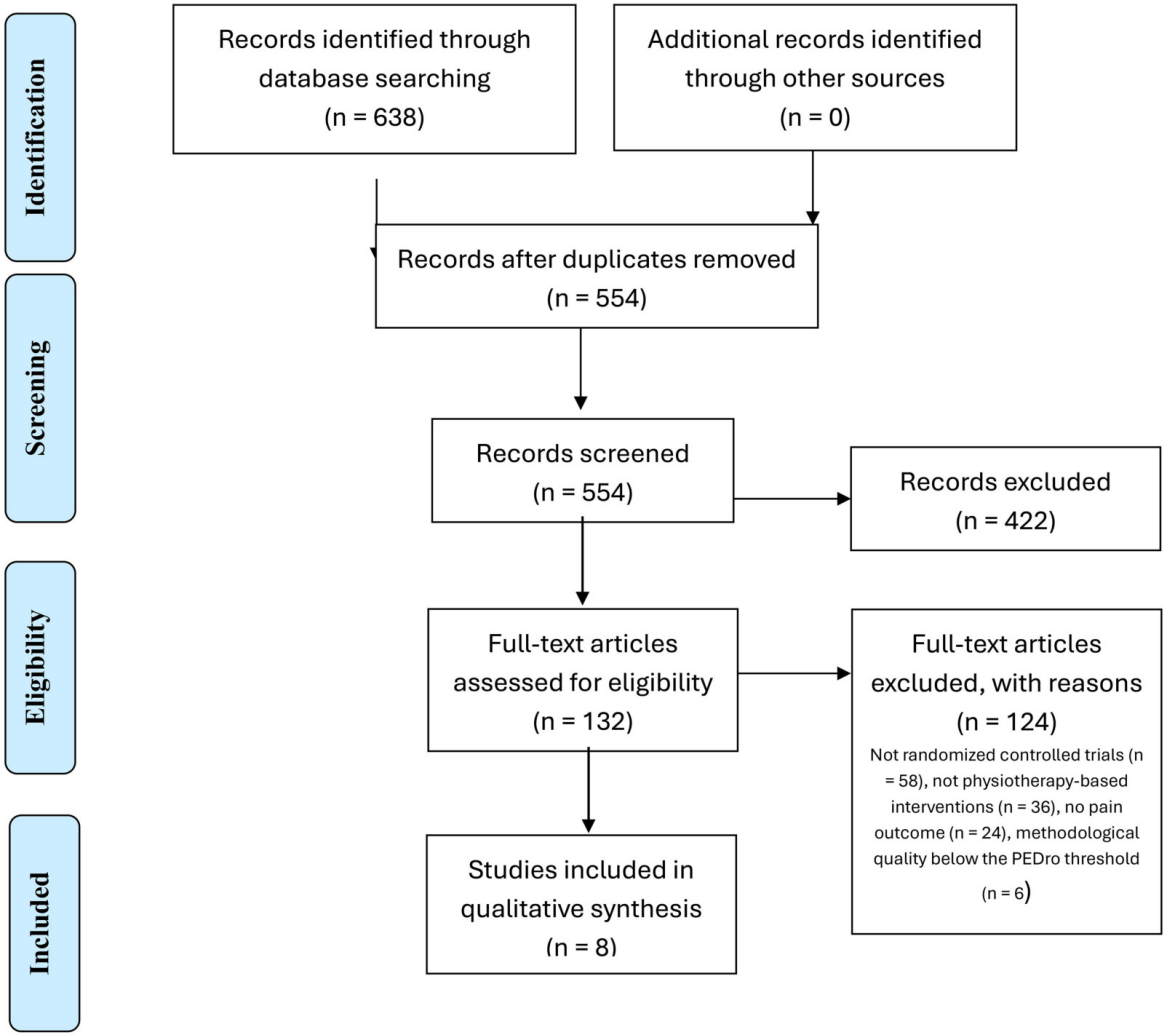
Flowchart of the study following PRISMA standards.

Figure 1 shows the flow diagram with the selection made according to the criteria described above.

### 4.2 Publications retrieved

#### 4.2.1 Summary of articles

Table 2 summarizes the main characteristics of the included studies, including sample size, intervention type, duration, outcomes assessed, and principal findings.

**Table 2:** Summary table of articles.

Author and Year	Design	Population	Variables (instrument)	Intervention	Results	MCID
Chan et al. (29)	Single-blind RCT	55 patients with colorectal cancer undergoing chemotherapy	Functionality (FACT), Quality of life (EORTC QLQ-C30), Pain (NRS), CIPN (FACT/GOG-Ntx), Fatigue	Group 1: Electroacupuncture 1x/week for 12 weeks. Group 2: Sham acupuncture	Significant improvements in functionality and reduction of CIPN symptoms	Not reported
He et al. (30)	Double-blind multicenter RCT	171 pancreatic cancer patients with abdominal/lumbar pain	Pain (NRS), Morphine use, Appetite, Constipation	Group 1: TENS 2x/day for 1 week. Group 2: Placebo	Significant decrease in pain without increase in morphine use	Not reported
Lu et al. (31)	Multicenter RCT with partial masking	576 patients scheduled for radical mastectomy	Chronic pain (NRS), Remifentanil use, Nausea/vomiting	Group 1: Transcutaneous electrical stimulation of preoperative acupoints at PC6 and CV17. Group 2: Transcutaneous electrical stimulation of acupoints on PC6. Group 3: Transcutaneous electrical stimulation of simulated acupoints	Reduction of chronic pain, more effective in combined acupoint stimulation	Not reported
Lyu et al. (32)	Double-blind RCT	159 patients with oncologic pain	Pain (NRS), Quality of Life (EQ-5D-3L), Karnofsky Performance Status (KPS) Morphine Use	Group 1: Transcutaneous electrical stimulation of acupuncture points 15 sessions in 3 weeks. Group 2: Transcutaneous electrical stimulation of sham acupoints	Statistically significant reduction of pain, without relevant clinical difference.	Clinically meaningful threshold predefined but not achieved
Müller et al. (34)	RCT with three arms	170 patients on platinum analogue chemotherapy	CIPN (TNSr and EORTC QLQ-CIPN15), Nerve conduction	Group 1: SMT. Group 2: RT. Group 3: Standard care	Sensory improvement only in patients adherent to exercise programs	Not reported
Song et al. (42)	Single-center placebo RCT	72 women with post-breast cancer CIPN	Pain (NRS), CIPN (TNS, EORTC QLQ-CIPN20), Quality of Life (FACT-B), and Pattern Identification (IPIE-CIPN)	Group 1: Portable electrostimulation on PC6. Group 2: Placebo	No significant differences were observed with placebo	Not reported
Tian et al. (33)	Controlled RCT	80 patients with advanced pancreatic cancer	Pain (NRS)	Group 1: Electrical stimulation at acupuncture points for 3 days. Group 2: Standard care	Sustained reduction of pain, without adverse effects	Not reported
Xiaoqian et al. (28)	Controlled RCT	108 patients with post-breast cancer CIPN	CIPN (FACT/GOG-Ntx), Quality of life (EORTC QLQ-C30), Physical function (6MWT)	Group 1: Compressive therapy + EXCAP. Group 2: Compressive therapy alone. Group 3: Control	Lower incidence of CIPN in groups with intervention; greater effect with combination	Not reported

RCT, randomized controlled trial; SMT, sensorimotor training; RT, resistance training; CIPN, chemotherapy-induced peripheral neuropathy; FACT/GOG-Ntx, functional assessment of cancer therapy/gynecologic oncology group–neurotoxicity questionnaire; EORTC QLQ-C30, European organisation for research and treatment of cancer quality of life questionnaire–core 30; FACT-F, functional assessment of cancer therapy–fatigue; FACT-B, functional assessment of cancer therapy–breast; TNSr, total neuropathy score (reduced version); NRS, numerical rating scale for pain; VAS, visual analog scale; DN4, douleur neuropathique 4 questionnaire; BFI, brief fatigue inventory; 6MWT, six-minute walk test; TEAS, transcutaneous electrical acupoint stimulation; TENS, transcutaneous electrical nerve stimulation; EA, electroacupuncture; KPS, Karnofsky performance status; EQ-5D-3L, EuroQol 5-dimension 3-level quality of life scale; UC, usual care; EXCAP, exercise for cancer patients program; PC6, pericardium 6 acupoint; CV17, conception vessel 17 acupoint; ST36, zusanli acupoint; SP6, sanyinjiao acupoint; L14, hegu acupoint.

#### 4.2.2 Assessment of heterogeneity, bias, and evidence quality

The randomized clinical trials included in this review showed notable methodological heterogeneity, both in the types of physiotherapeutic interventions evaluated (e.g., electrical stimulation, therapeutic exercise, and compressive therapy) and in their frequency, duration, and outcome variables. Because of these differences, a quantitative meta-analysis was not appropriate, and a systematic review approach was adopted. The risk of bias of the included studies was assessed using the Cochrane Risk of Bias 2.0 (RoB 2.0) tool, and their methodological quality was evaluated with the PEDro scale. Overall, the studies presented low to moderate risk of bias and satisfactory methodological quality, with PEDro scores ranging from 6 to 10, supporting the reliability of their findings despite the heterogeneity of designs and measures.

#### 4.2.3 Description of the population

The present systematic review included eight randomized clinical trials (RCTs), with a total of 514 participants. The populations studied were heterogeneous, although all shared oncologic diagnosis as a common criterion. Most of the studies focused on patients with colorectal cancer and breast cancer, followed by cases with advanced pancreatic cancer and other types associated with treatments with neurotoxic agents, such as platinum analogues.

Regarding sociodemographic characteristics, several studies included exclusively women (27, 28), while others incorporated mixed populations, such as Chan et al. (29) or He et al. (30). Ages ranged from approximately 40–70 years, although they were not homogeneously reported. In all cases, participants had symptoms of cancer-related pain, CIPN, or were at risk of developing chronic postoperative pain. Across the included randomized controlled trials, CIPN was the most frequently investigated pain condition, accounting for the majority of participants and outcome data synthesized in this review.

#### 4.2.4 Description of the interventions and comparators applied

The physiotherapeutic techniques applied were grouped into three main categories:

Electrical stimulation (TEAS, TENS, electroacupuncture): applied in six studies, at specific acupoints, with frequencies ranging from 2 Hz to 100 Hz, customized intensities and sessions ranging from a single weekly intervention for 12 weeks to intensive treatments of 15 sessions in 3 weeks (27, 29–33).
Therapeutic exercise and neuromuscular training: used in the studies of Müller et al. (34) and Xiaoqian et al. (28). Structured protocols of resistance training (RT), sensory motor therapy (SMT), progressive aerobic walking (EXCAP) and elastic band exercises were applied. In the study by Müller et al. (34), participants underwent a 12-week combined RT–SMT program performed three times per week, targeting improvements in muscle strength, coordination, and sensory function in patients with CIPN. The resistance component consisted of progressive multi-joint exercises (e.g., squats, leg press, and seated rowing) at moderate intensity, adjusted according to tolerance, while SMT included balance and proprioceptive tasks on unstable surfaces. Significant improvements were reported in vibration sensitivity and functional mobility scores in patients with high adherence.
In the trial by Xiaoqian et al. (28), a combined compression and exercise protocol was implemented to prevent and manage CIPN. The exercise regimen followed the EXCAP model (Exercise for Cancer Patients), consisting of daily walking and progressive home-based aerobic activity complemented by light resistance training using elastic bands. The intervention group receiving combined compression therapy and EXCAP showed a lower incidence of CIPN, better six-minute walk test performance, and higher quality-of-life scores compared with control participants.
Compressive therapy: implemented in combination or in isolation in the study by Xiaoqian et al. (28) with the aim of preventing or reducing the incidence of CIPN during chemotherapy infusion.

The control groups received sham interventions (such as sham acupuncture or placebo stimulation) or usual care. Ethical principles were respected in all studies, and the interventions were considered safe, non-invasive and well tolerated.

#### 4.2.5 Description of the outcome variables analyzed

The variables assessed in the studies were heterogeneous, but shared a common focus on addressing physical symptoms derived from cancer treatment. The most frequently analyzed were:

Pain: present in 7 of the 8 studies, assessed using scales such as the NRS (Numeric Rating Scale), VAS and clinical reports of analgesic use.
Peripheral neuropathy: specifically assessed in studies with patients treated with oxaliplatin or taxanes, using tools such as FACT/GOG-Ntx, functional assessments and subjective sensory symptoms.
Quality of life: addressed in a complementary manner in studies such as Chan et al. (29) and Lyu et al. (32).
Physical function, fatigue, nausea, vomiting and appetite: also reported as secondary variables in surgical or palliative settings.

To a lesser extent, electrophysiological parameters (nerve conduction, muscle action potential) and opioid use were assessed as proxy indicators of therapeutic efficacy

#### 4.2.6 Description of individual studies

Chan et al. (29) reported that weekly electroacupuncture for 12 weeks significantly reduced CIPN symptoms and improved physical function, with good tolerability and no notable changes in sensitivity testing.
He et al. (30) found that TENS significantly decreased abdominal and back pain in patients with pancreatic cancer, with no increase in morphine use and secondary improvements in appetite and constipation.
Lu et al. (31) demonstrated that TENS applied before mastectomy reduced the incidence of chronic pain at 6 months, with combined acupoint stimulation being more effective than single-point stimulation.
Lyu et al. (32) documented a statistically significant reduction in pain after 3 weeks of TEAS, although the magnitude of change did not reach a clinically significant difference.
Müller et al. (34) evaluated two exercise programs and found that, although intention-to-treat analysis evidenced no difference, those patients adherent to the protocols did report less increase in CIPN sensory symptoms.
Song et al. (27) found no significant differences in CIPN symptoms between the portable electrostimulation group and placebo, although they suggested possible therapeutic effects on pain reduction.
Tian et al. (33) observed consistent improvements in pain severity with transcutaneous acupoint stimulation in patients with advanced pancreatic cancer, with no relevant adverse effects.
Xiaoqian et al. (28) concluded that both compressive therapy and its combination with EXCAP progressive exercise reduced the incidence of CIPN, with the combined intervention being more effective.

Across the eight randomized clinical trials included, physiotherapeutic interventions produced beneficial effects in several key outcomes. Pain intensity decreased significantly in studies that applied transcutaneous electrical stimulation or electroacupuncture techniques (30–33). (CIPN symptoms improved in trials that implemented structured exercise programs or compressive therapy (28, 34), whereas quality of life and functional capacity showed measurable gains in interventions combining electrostimulation or progressive exercise (29, 32). One study (27) reported no significant differences compared with placebo. Collectively, these findings indicate that physiotherapy-based interventions can reduce pain and neuropathic symptoms while enhancing function and quality of life in oncology patients.

#### 4.2.7 Bias analysis of the study articles

**Figure 2:**
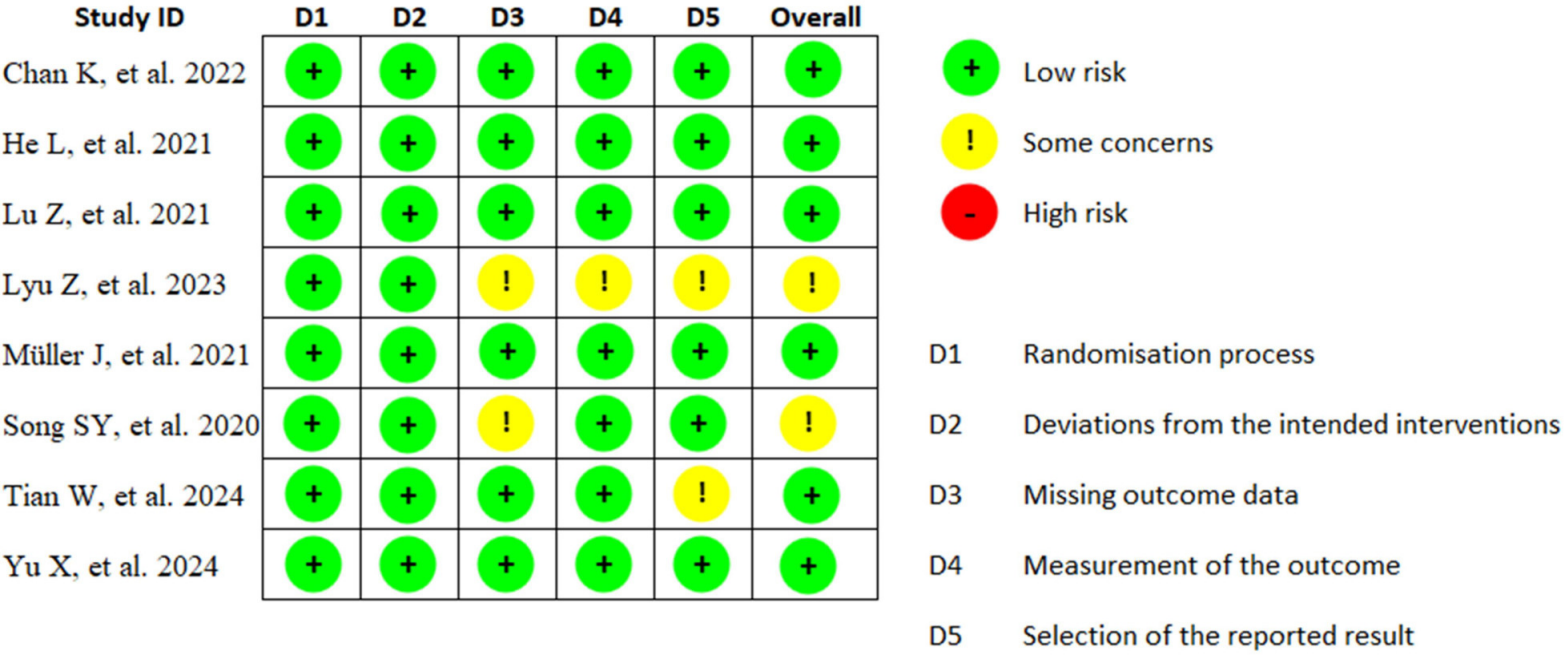
Analysis of the methodological biases of the studies analyzed.

Figure 2 shows the analysis of the methodological biases of the studies analyzed in this review.

#### 4.2.8 Qualitative analysis of the level of scientific evidence

Assessment of the level of scientific evidence of the selected articles can be appreciated through Table 1.

Using a simplified GRADE-informed narrative approach, the overall certainty of evidence across intervention categories was judged to be low. Exercise-based rehabilitation interventions were supported by low-certainty evidence, primarily due to small sample sizes, heterogeneity of exercise protocols, and indirectness related to chemotherapy-induced peripheral neuropathy–specific populations. Electro-physical agent interventions, including transcutaneous electrical stimulation and electroacupuncture, were also supported by low-certainty evidence, reflecting methodological limitations, variability in intervention parameters and outcome measures, and inconsistent effects across trials. Evidence for combined or multimodal interventions was similarly rated as low certainty because of the limited number of studies and exploratory trial designs.

## 5 Discussion

The present systematic review focused on the available scientific evidence on the efficacy of different physiotherapeutic interventions in the management of cancer-related pain. The results show that techniques such as therapeutic exercise, electrical stimulation, manual lymphatic drainage and myofascial therapy have positive results in pain relief and improvement of quality of life (15). Although this review addresses cancer-related pain broadly, the current randomized evidence is largely concentrated on CIPN, a predominantly neuropathic pain condition. As a result, the observed effects of physiotherapy-based interventions should be interpreted primarily within the context of neuropathic pain following chemotherapy exposure. Evidence regarding other cancer-related pain mechanisms, including nociceptive, visceral, or bone metastasis–related pain, remains limited and cannot be inferred from the present findings.

When compared with previous systematic reviews and meta-analyses, our findings are broadly consistent with the growing body of evidence supporting the therapeutic role of physiotherapy and structured exercise in oncology. Recent meta-analyses by Guo et al. (16) and Amarelo et al. (9) concluded that physical exercise interventions may significantly reduce the severity of chemotherapy-induced peripheral neuropathy and improve patients' functional outcomes, although both emphasized the methodological heterogeneity among included trials. Likewise, Peters et al. (23) reported that physical activity programs contribute to pain reduction in people with cancer, while qualitative synthesis by Burke et al. (22) highlighted that exercise enhances quality of life, self-efficacy, and psychological well-being among cancer survivors. Our review complements these earlier analyses by incorporating more recent RCTs (2020–2024) and a wider spectrum of physiotherapeutic techniques, including electrical stimulation and compression therapy.

Furthermore, high-quality studies such as the international consensus by Campbell et al. (35), and recent clinical trials by Fernández-Rodríguez et al. (36) and Lin et al. (37) reinforce the positive direction of evidence toward the safety and potential efficacy of multimodal physiotherapy interventions in pain and functional outcomes. Although methodological heterogeneity precluded a meta-analytic synthesis, the convergence of findings across these studies supports the growing clinical relevance of physiotherapy within multidisciplinary cancer care. The exercise modalities identified in this review correspond closely to the principles established in current evidence-based guidelines and clinical research. The Exercise Guidelines for Cancer Survivors (35) recommend that cancer patients engage in a combination of aerobic and resistance training of moderate intensity, performed two to three times per week and individualized to each patient's capacity, treatment phase, and symptom burden. These recommendations are grounded in the concept that exercise is medicine—that is, exercise should be prescribed with the same clinical precision and monitoring as pharmacological therapy.

This approach is supported by recent randomized clinical trials indicating the possible effectiveness and safety of structured, multimodal exercise interventions across different oncological populations. For example, Fernández-Rodríguez et al. (36) reported significant improvements in functional autonomy, fatigue, and overall quality of life following a supervised rehabilitation program combining aerobic and resistance exercises in hospitalized oncology patients. Similarly, Lin et al. (37) showed that programs integrating joint-mobility, aerobic, and progressive-resistance training under structured follow-up significantly improved pain, prevented lymphedema, and enhanced long-term quality of life in breast-cancer survivors.

Taken together, these studies reinforce the clinical rationale for viewing exercise therapy not as a generic adjunct but as a therapeutic prescription that must be personalized, supervised, and progressively adapted to the patient's clinical status.

In our review, five of the eight studies employed electrical stimulation techniques (TENS, TEAS, electroacupuncture), which showed mostly positive results in the reduction of oncologic pain. These interventions coincided with that reported by Stout et al. (38), who highlighted improvements in functional mobility and quality of life in a high percentage of oncologic rehabilitation studies. However, some trials in our sample, such as those by Lyu et al. (32) and Song et al. (27) did not report clinically significant differences with respect to placebo, which reinforces the need for greater Methodological precision and standardization of therapeutic parameters, as had already been pointed out in previous reviews.

On the other hand, the use of therapeutic exercise, especially in combination with other interventions such as compressive therapy (28) showed benefits in the reduction of neuropathic pain, particularly CIPN. These findings align with the conclusions of Guo et al. (16) and Amarelo et al. (9) who highlighted the potential of exercise to mitigate neuropathic symptomatology. In turn, the study by Müller et al. (34) in our review evidenced significant sensory improvements in patients adhering to sensorimotor exercise programs, which coincides with that indicated by Dennett et al. (39) regarding the improvement of physical function and muscle strength, although the latter group of authors also warned about the paucity of evidence regarding impacts on the overall health system.

It is striking that, despite the emerging positive evidence, a structural invisibility of physiotherapy within oncology plans persists, as underlined by Brennan et al. (40). This institutional gap may also have conditioned the low number of studies with high Methodological quality available, a limitation that our own review has evidenced.

Furthermore, although some interventions such as acupuncture, auriculotherapy and cryotherapy were not included in our review due to eligibility criteria, observational studies and reviews conducted in Latin American contexts (41) reinforce their potential clinical value. Although they cannot yet be considered first-line interventions, their progressive incorporation as complementary merits attention in future research.

Overall, our findings agree with much of the international literature regarding the benefits of exercise and other physiotherapeutic techniques in the management of oncologic pain, but also highlight the fragmentation of the approach, the need to standardize protocols and to expand the evidence on specific types of pain such as neuropathic pain. Oncologic physical therapy has indisputable clinical potential, but its consolidation in practice requires adequate institutional frameworks, rigorous research and better integration within the oncologic interdisciplinary team.

### 5.1 Limitations of the review

Heterogeneity in study designs: The studies included in this review presented considerable variability in their methodological designs, which limits the ability to make direct comparisons between different interventions.

Diversity in interventions: The physical therapy techniques employed varied widely in terms of modalities, intensities, frequencies, and durations. For example, both transcutaneous electrical stimulation and acupuncture techniques were employed, each with different protocols. This lack of standardization limits the ability to determine which modality is most effective in the treatment of cancer-related pain, and comparability across studies is compromised. However, given that the majority of included studies focused on this specific neuropathic pain condition, conclusions should not be generalized to all forms of cancer-related pain. Further high-quality trials are needed to evaluate the role of physical therapy across other cancer-related pain mechanisms and clinical contexts.

Limited sample size and statistical power: Some of the included studies had relatively small samples, which may have influenced the statistical power of the results and the generalizability of the findings to a broader population of oncology patients. Although several studies showed positive results, some did not reach statistical significance due to sample size limitations.

### 5.2 Relevance to clinical practice

The findings of this review support the use of noninvasive physical therapy interventions as part of the comprehensive management of cancer-related pain and chemotherapy-induced neuropathy. Techniques such as TEAS, TENS and electroacupuncture can be implemented in various clinical settings, offering a safe and accessible alternative to pharmacological treatments. Likewise, therapeutic exercise programs, especially when combined with measures such as compressive therapy, can contribute to the prevention of sensory and functional complications. These results reinforce the active role of the physiotherapist in the multidisciplinary approach to the oncology patient, highlighting the importance of individualizing interventions according to the type of cancer, stage of treatment and general condition of the patient.

## 6 Future lines of research

In terms of future lines of research, several key areas are identified as requiring further attention. First, the need to standardize intervention protocols is highlighted, as there is currently considerable heterogeneity in terms of parameters such as frequency, duration, intensity and points of application of the techniques used. In addition, most of the included studies evaluated short-term effects, so it is recommended that trials with prolonged follow-ups be developed to analyze the sustainability of clinical benefits. It is also fundamental to broaden the spectrum of variables analyzed in future studies, extending beyond pain intensity to include measures such as fatigue, functional capacity, psychological well-being, and long-term quality of life. Evaluating these multidimensional outcomes would provide a more comprehensive understanding of the overall impact of physiotherapeutic interventions on cancer survivorship.Another relevant aspect is the inclusion of more representative and diverse samples, both in terms of types of cancer and demographic and socioeconomic factors. Finally, it would be valuable to design comparative studies between different physiotherapeutic interventions, as well as research that explores the synergistic effect of therapeutic combinations, including cost-effectiveness and adherence analyses.

## 7 Conclusions

This systematic review suggests that selected physiotherapy-based interventions, including electrotherapeutic modalities and therapeutic exercise, may offer safe and potential benefits for pain-related and functional outcomes in individuals with CIPN. The available randomized evidence predominantly addresses neuropathic pain, and findings should therefore not be generalized to other cancer-related pain phenotypes. Despite methodological heterogeneity across studies, physiotherapy interventions may represent valuable complementary options, particularly when pharmacological management is insufficient or poorly tolerated. These findings reinforce the active role of the physiotherapist in the oncology team and highlight the need for more rigorous research to consolidate these interventions as an essential part of the comprehensive approach to the oncology patient, promoting a person-centered and optimal care, aimed at improving their quality of life.

## Data Availability

The original contributions presented in the study are included in the article/Supplementary Material, further inquiries can be directed to the corresponding author.

## Annex 1 Complete search strategy.

**Table:**

Database	Search Equation
PubMed	((((((("physiotherapy"[MeSH Terms]) OR ("physical therapy"[MeSH Terms])) OR ("exercise therapy"[MeSH Terms])) OR ("manual therapy"[Title/Abstract])) OR ("rehabilitation"[MeSH Terms])) OR ("oncological rehabilitation"[Title/Abstract]) OR ("cancer rehabilitation"[Title/Abstract])) AND (("cancer patients"[Title/Abstract]) OR ("cancer survivors"[Title/Abstract]))) AND (("pain management"[MeSH Terms]) OR ("lymphedema management"[Title/Abstract]) OR ("fatigue management"[Title/Abstract]) OR ("quality of life"[MeSH Terms]) OR ("electrotherapy"[MeSH Terms]) OR ("breathing exercises"[Title/Abstract])))
Cochrane Central Register of Controlled Trials (CENTRAL)	(physiotherapy OR "physical therapy" OR "exercise therapy" OR "manual therapy" OR rehabilitation OR "oncological rehabilitation" OR "cancer rehabilitation") AND ("cancer patients" OR "cancer survivors") AND ("pain management" OR "lymphedema management" OR "fatigue management" OR "quality of life" OR electrotherapy OR "breathing exercises")
CINAHL	t:("physiotherapy") OR t:("physical therapy") OR t:("exercise therapy") OR t:("manual therapy") OR t:("rehabilitation") OR t:("oncological rehabilitation") OR t:("cancer rehabilitation") AND t:("cancer patients") OR t:("cancer survivors") AND t:("pain management") OR t:("lymphedema management") OR t:("fatigue management") OR t:("quality of life") OR t:("electrotherapy") OR t:("breathing exercises")
PEDro	physiotherapy OR "physical therapy" OR "exercise therapy" OR "manual therapy" OR rehabilitation OR "oncological rehabilitation" OR "cancer rehabilitation" AND "cancer patients" OR "cancer survivors" AND "pain management" OR "lymphedema management" OR "fatigue management" OR "quality of life" OR electrotherapy OR "breathing exercises"
